# Bis(2‐amino‐5‐thienyl)Ketone as Oxygen Tolerant Sensitizer for Conventional Radical Photopolymerization

**DOI:** 10.1002/anie.202518608

**Published:** 2025-12-16

**Authors:** Taner Poplata, Qunying Wang, Xavier Allonas, Martin Jäger, Jochen S. Gutmann, Evgenia Dmitrieva, Horst Hartmann, Bernd Strehmel

**Affiliations:** ^1^ Institute for Coatings and Surface Chemistry Department of Chemistry Niederrhein University of Applied Sciences Adlerstr. 1 47798 Krefeld Germany; ^2^ Laboratory of Macromolecular Photochemistry and Engineering Université de Haute Alsace 3b rue Alfred Werner Mulhouse 68093 France; ^3^ Department of Physical Chemistry and Center of Nanointegration (CENIDE) University of Duisburg‐Essen Universitätsstr. 7 45151 Essen Germany; ^4^ Leibniz Institute for Solid State and Materials Research (IFW) Dresden Helmholtzstr. 20 01069 Dresden Germany; ^5^ Faculty of Chemistry and Food Chemistry Technical University Dresden 01062 Dresden Germany

**Keywords:** Bis(2‐amino‐5‐thienyl) ketones, Crosslinking, Low cytotoxic, Oxygen tolerance, Radical photopolymerization

## Abstract

Bis(2‐amino‐5‐thienyl)ketones (**TK**s) operated as effective sensitizer in a multi‐component photoinitiating system for conventional radical photopolymerization. The distinct amino pattern (morpholino **1** versus diaryl moiety **2**) followed an oxidative photoinduced electron transfer (**PET**) mechanism with the iodonium salt **C1** and the sulfonium cation **C2**. **2** performed well under air upon UV‐LED exposure at 395 nm. The **TK**s mostly reacted from the triplet state (T_1_) with either **C1** or **C2** resulting in a cation radical with certain instability. This changes considering the radical anion studied by spectroelectrochemistry confirmed by reversibility. Electrochemical measurements and transient absorption experiments disfavor from a thermodynamic point of view a reductive mechanism of the T_1_ with **C3**. Long‐lived charge separated states discuss a new paradigm as a further alternative to form reactive intermediates even with **C3**. ^1^O_2_ generation by ^3^ TK* operated as additional pathway to form the initiating ∼N─CH_2_• radical of **C3**. The higher efficiency of photoinduced electron transfer (**PET**) with **C1** explained the higher network density of the crosslinked material. The subsequent [4+2] cycloaddition of ^1^O_2_ to the thiophene moiety led to a mass increase of 32 *m_z_* of either **1** or **2**, which led to depletion of inhibiting ^3^O_2_. This additionally explains the unexpected, good oxygen tolerance. Furthermore, **TK 2a** showed no significant cytotoxic response explaining its future potential for biocompatible applications.

## Graphical abstract

Bis(2‐amino‐5‐thienyl)ketones operate as sensitizer in multi‐component photoinitiating systems using either diaryl iodonium cation or amino benzoates as coinitiators to initiate conventional radical polymerization following either oxidative or reduction photoinduced electron transfer protocol, respectively. These systems result in formation of crosslinked polymers even under aerobic conditions. A 395 nm emitting LED operated as exposure source.

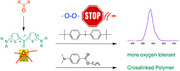

## Introduction

Light‐mediated conventional radical polymerization has received significant attention as a green technology^[^ ^1^, ^2^ ^]^ due to its numerous applications in imaging^[^ ^3^, ^4^, ^5^ ^]^ 2D printing,^[^ ^6^, ^7^ ^]^ 3D printing,^[^ ^8^, ^9^, ^10^, ^11^, ^12^ ^]^ dentistry,^[^ ^13^ ^]^ orthopedics,^[^ ^8^ ^]^ coatings,^[^ ^14^, ^15^, ^16^, ^17^, ^18^ ^]^ and lithography,^[^ ^19^ ^]^ among others. Photopolymerization has continuously developed even with no discontinuity in the pandemics^[^ ^20^ ^]^ due to the strong requirements addressed by new applications. Conventional radical polymerization (IUPAC decided to discontinue the use of the term free radical polymerization^[^ ^21^ ^]^) has mostly attracted transfer to practice while light‐mediated controlled polymerization has been still waiting to make the first big step although first attempts were almost reported.^[^ ^22^, ^23^ ^]^

One or multi‐component photoinitiating systems, formerly called as Type I and Type II systems,^[^ ^24^ ^]^ responsibly generate initiating radicals. One‐component systems generate initiating radicals. This proceeds by either bond cleavage in a Norrish I protocol^[^ ^1^ ^]^ or single bond cleavage in Iodonium salts by UV‐LED exposure^[^ ^25^ ^]^ while multi‐component systems require participation of at least reactant. Generation of initiating radicals occurs by photoinduced electron transfer^[^ ^1^ ^]^ (**PET**) or bond cleavage of sterically hindered bisimidazoles reacting in a consecutive step with mercapto compounds.^[^ ^1^, ^26^ ^]^ **PET** based systems comprise at least a sensitizer **Sens** and a coinitiator. This principle, introduced many decades ago,^[^ ^27^, ^28^, ^29^, ^30^, ^31^ ^]^ where thermodynamic parameters such as the oxidation and reduction potentials of the participating reactants importantly tune the Gibbs energy of electron transfer Δ*G*_el_ following either an oxidative or reductive **PET**.^[^ ^32^ ^]^ The biggest benefit of multi‐component photoinitiating systems can be seen to operate them from the visible to the near‐infrared (NIR) region.^[^ ^1^, ^16^, ^18^, ^33^, ^34^, ^35^ ^]^ Here, they present alternatives to one‐component systems whose absorption mostly finishes above 400 nm.^[^ ^1^, ^36^ ^]^ Recently, toxicological issues appeared regarding the one‐component photoinitiators **BAPO** and **TPO**.^[^ ^37^, ^38^, ^39^ ^]^

Multi‐component systems can additionally operate based on a photocatalytic mechanism enabling controlled photopolymerization (photo‐RAFT,^[^ ^34^, ^40^, ^41^ ^]^ photo‐ATRP^[^ ^34^, ^42^, ^43^, ^44^, ^45^, ^46^, ^47^, ^48^ ^]^) or conventional radical photopolymerization with regeneration of the sensitizer by adding of a respective donor whose **HOMO** energy remains above the **SOMO** energy of **Sens** ^+•^ while the LUMO of the acceptor is below the **SOMO** of **Sens^−^** ^•^.^[^ ^7^, ^30^, ^49^ ^]^

Alternatively, generation of initiating radicals based on conventional radical polymerization with two components connected with bleaching of **Sens**.^[^ ^50^, ^51^ ^]^ The latter has received the largest acceptance regarding its transfer to praxis where curing of samples with large thickness represents the major challenge.^[^ ^36^, ^51^ ^]^ Here, the photochemistry of ketones attracted attention^[^ ^52^ ^]^ explaining their use in many applications.^[^ ^1^ ^]^ Benzophenone (**BP**) comprising materials represented one of the “running horses”.^[^ ^1^, ^52^, ^53^ ^]^

The technological development additionally required to search for structures exhibiting a bathochromic shifted absorption to enable them with more technologies.^[^ ^1^ ^]^ Different aromatic ketones received additional attention explaining their enhanced use in practice.^[^ ^1^ ^]^ This also required a more detailed understanding of photophysical properties as shown previously in a comparative study focusing on an oxidative **PET** with diaryl iodonium cations as coinitiator.^[^ ^54^ ^]^ Particular bridged ketones, such as thioxanthones, have received big application potential in multicomponent photoinitiating systems.^[^ ^1^ ^]^ However, the large distribution and the increased number for uses of them in technologies related to packaging, also those related to food packaging, has shown toxicological issues caused by aromatic ketones, particular thioxanthones.^[^ ^55^ ^]^ Thus, a demand exists for alternatives.

Photoinitiating systems also operate as heterogeneous components.^[^ ^2^ ^]^ This has received gained interest, accelerated by the introduction of carbon nanodots **CD**s.^[^ ^56^, ^57^ ^]^ Different functionalization strategies such as the functionalization in an organic network,^[^ ^58^ ^]^ and confinement in different matrices^[^ ^59^ ^]^ increased the performance. Recently, a new one‐component photoinitiator has brought new impetus in this field.^[^ ^60^ ^]^ These materials can be seen as less toxic^[^ ^60^ ^]^ and may become an alternative in future.

Despite these positive developments,^[^ ^20^ ^]^ there still exist additional requirements. First, radical photopolymerization under air appears as challenging although promising attempts were introduced to deactivate inhibiting oxygen. This can be singlet oxygen generators^[^ ^61^ ^]^ or a protocol based on breathing photo‐ATRP by adding pyruvate.^[^ ^62^, ^63^ ^]^ Practice demands to provide more oxygen tolerant systems, which still appears as lacking. Second, toxicological issues represent a further topic. Frequently, more reports appeared around the toxicity of phosphine oxide based photoinitiators.^[^ ^37^, ^38^, ^39^ ^]^ Here, tetraacylstannanes could be an alternative due to their low cytotoxicity^[^ ^64^, ^65^ ^]^ but their use has been limited to a few applications. Here, heterogeneous materials appeared as interesting alternatives^[^ ^2^, ^60^, ^66^ ^]^ but their transfer to practice has been still in progress. Nowadays, the use of strong radiators helps to overcome oxygen inhibition. This often requires to operate with Hg lamps whose use will be questionable from 2027^[^ ^67^ ^]^ though some exceptions may extend this shift (see document in chapter 7 of Supporting Information).

Surprisingly, materials comprising heterocyclic moieties such as thiophene have not received the necessary attention. They can sometimes contribute to detoxification of the organism.^[^ ^68^, ^69^ ^]^ In addition, they build in some cases flavoring ingredients in foods.^[^ ^70^ ^]^ Thus, such structural patterns could receive additional audience regarding the development of photosensitizers with less toxicological concern.

**Scheme 1:**
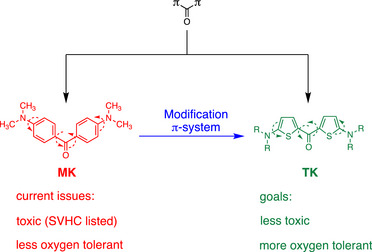
Structural comparison between Michlers Ketone (**MK**) and thiophene based aromatic ketones (**TK**).

This has motivated to search for sensitizers (**Sens**) selected from aromatic ketones comprising π‐CO‐π, with π as aromatic moiety.^[^ ^54^ ^]^ Here, the photophysical properties of Michler's ketone (**MK**), for example, served as one fundamentals to search for alternative structural patterns. Its sensitizing properties to generate initiating radicals brought the attention to photopolymer science^[^ ^54^ ^]^ while it can be found on the “SVHC” list recently.^[^ ^71^ ^]^ It also does not tolerate oxygen in practical systems. Thus, bis(2‐amino‐5‐thienyl)ketones (**TK**) (Scheme 1) exhibit a pattern similar to that of in aromatic ketone π‐CO‐π. They mainly differ regarding the π structure of the aromatic moiety while both, **MK** and **TK**, comprise flexible bonds at the carbonyl and amino moiety. Scheme 1 illustrates the scenario. The molecular structure of **TK**s compared to **MK** ^[^ ^54^ ^]^ shifts their absorption bathochromic enabling even blue light emitting LEDs operated for some biomedical applications.^[^ ^1^ ^]^

These challenges require to study the photophysical properties to explore the function of the excited state, which can be the first excited singlet (S_1_) or triplet state (T_1_). This furthermore questions whether the reaction follows an oxidative or reductive **PET** where particular the thiophene moiety enables efficient photopolymerization under air. This feature additionally motivated to transfer these materials to practical applications.^[^ ^72^ ^]^

## Results and Discussion

### Selection of Materials

**Scheme 2:**
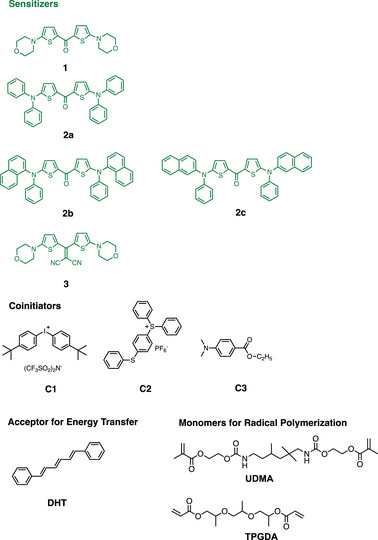
Summary of Structures used.

Scheme 2 exhibits the respective structures, whose synthesis were described earlier.^[^ ^73^, ^74^, ^75^, ^76^ ^]^ Its substitution with aliphatic amines such as morpholine yielded **1** while introduction of aromatic amine moieties resulted in  **2a**‐**c**. Additional reaction of **1** with malononitrile yielded **3**, which should in contrast to **1** preferentially operate from the S_1_.

Thus, **1** and **2a**‐**c** should preferentially operate as sensitizer (**Sens**) from the T_1_ in a photoinduced electron transfer (**PET**) protocol where the electron back transfer proceeds less efficient in comparison with those operating mainly from the S_1_. In addition, efficient formation of the T_1_ requires to consider singlet oxygen (^1^O_2_) formed by energy transfer between the T_1_ and ^3^O_2_. ^1^O_2_ results in the less inhibiting superoxide radical,^[^ ^61^ ^]^ explaining a certain oxygen tolerance. In addition, the thiophene moieties can add ^1^O_2_ following a [4+2] cycloaddition.^[^ ^77^ ^]^ This reaction also contributes to the conversion of inhibiting triplet oxygen ^3^O_2_ into non‐inhibiting products, as it is initially chemically bound in this mechanism.

Furthermore, the absorption of **1**–**3** covers the UV, blue, and, depending on substitution pattern, also parts of the green spectral region. This would enable their use in different technologies depending on the respective spectral emission of the light source. 2D printing,^[^ ^6^ ^]^ 3D printing,^[^ ^9^, ^11^, ^49^, ^78^, ^79^ ^]^ and some holographic patterning techniques may mainly benefit from this feature.^[^ ^3^, ^5^ ^]^

The heterocyclic thiophene structure should be associated with less toxic issues connecting to fewer toxic properties. Those heterocycles could detoxicate the metabolism resulting in a less toxic risk following a proposal disclosed earlier.^[^ ^69^ ^]^ Thus, these structures would be interesting alternatives to Michler's ketone (**MK**) whose sensitizing property in multi‐component photopolymer systems was already demonstrated including the performance.^[^ ^52^, ^54^ ^]^ However, Michler's ketone was disclosed as toxic. Its further use in photopolymerization systems can be seen as critical. REACH classified **MK** to the list of substances of very high concern (“SVHC” list).^[^ ^71^ ^]^ A negative approval of the best candidate shown in Scheme 2 could be seen as a huge benefit in the field of multi‐component photoinitiating materials since alternatives such as *i*‐propyl thioxanthone (**ITX**) caused problems in daily applications.^[^ ^39^, ^55^ ^]^ The lower toxicity of the sensitizers disclosed can be seen as an additional benefit compared to one‐component photoinitiators such as **TPO** or **BAPO** and two‐component initiators such as **ITX**. Here, toxicity issues have been increasing in the last years.^[^ ^37^, ^38^, ^39^, ^55^, ^69^ ^]^

### Electrochemical and Spectroelectrochemical Measurements

The cyclic voltammograms (CVs) of **1** und **2a**‐**c** measured in acetonitrile show one reversible reduction and two or more irreversible oxidation events (see Supporting Information). The reduction potentials appear in the potential range from −1.94 to −2.21 V (versus Fc/Fc^+^) indicating that reduction of the molecules does not easily proceed. However, the reduction potential of diamino thienyl‐substituted ketones appears significantly higher than that of **MK** (‐2.62 V). An irreversible oxidation with peak potential at 0.4–0.52 V points the instability of the radical cation addressing this species to undergo consecutive reactions. The oxidation potentials of **TK**s are comparable with that of **MK** (0.57 V). For comparison, data of **MK** were retaken to avoid any irritation to previous data.^[^ ^80^ ^]^ Figure S1 shows the cyclic voltammograms of the **TK**s and **MK**.

**Figure 1:**
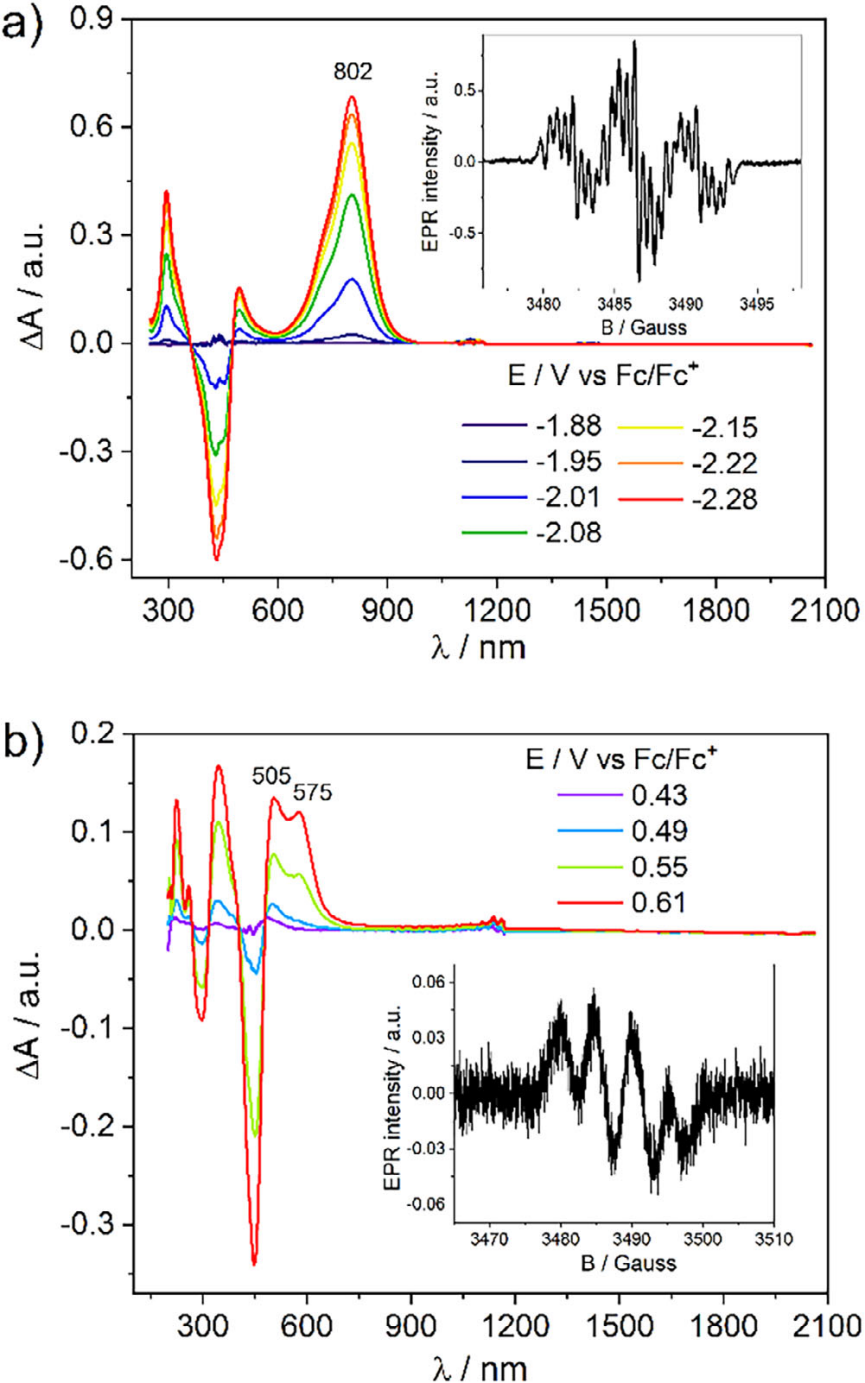
In situ UV–Vis–NIR absorption spectra of **2a** measured during the reduction a) and oxidation b) process. Inset: EPR spectrum of radical anion **2a** ^•−^ and radical cation **2a** ^•+^.

The reversible reduction behavior of the compounds enables to perform in situ EPR/UV–vis–NIR spectroelectrochemistry. The EPR spectra of the bis(2‐amino‐5‐thienyl)ketones exhibit a signal with a *g* value of 2.0044 and a hyperfine structure (Figure 1a, see more information vide infra). The largest hyperfine splitting coupling (*hfc*) constants (*a*(^1^H) = 4.3–4.8 Gauss) originate from two equivalent β‐protons in the thiophene rings near the oxygen (1:2:1 triplet) (Table in S1). The additional splitting relates to the other β‐protons in the thiophene rings (ca. 1 Gauss) and nitrogen nuclei (∼0.6 Gauss). This indicates delocalization of the spin density in the radical anion across both amino thiophenes, with a significant contribution located near the oxygen. The UV–vis–NIR absorption spectra of the radical anions show an intense band in the range of 767–825 nm (see Figure S2 and Table S1). The position of the band slightly red‐shifts in the row **2a**<**2c**<**2b**. In the case of **1** that comprises morpholine as aliphatic amino moiety, the absorption band significantly blue‐shifts compared to that comprising the diphenylamino group (767 and 802 nm for **1** and **2a**, respectively).

During the oxidation, the EPR spectra of **2a** exhibit a weak signal with a *g* value of 2.0031 (Figure 1b). The *hfc* constants originate to one nitrogen atom and one β‐proton (5.2 and 4.0 Gauss, respectively) pointing that the spin density in the radical cation prefers localization on one side of the molecule. Thus, the spin density distribution in the radical anion and radical cation is completely different. This can explain the difference between their stabilities. The results found for the agree with those disclosed for **MK** where distorted structures were reported.^[^ ^80^ ^]^

### Photophysical Properties and Thermodynamic Considerations of PET

Photoinduced electron transfer can follow an oxidative or reductive mechanism^[^ ^32^ ^]^ where the reaction between the excited state of the sensitizer Sens* with an acceptor (**Ar_2_I** ^+^ **X** ^−^) and donor (**D**–**H**) results in formation of the radicals **Ar_2_I** ^•^ + **D** ^•^, respectively. Equations 1 and 2 disclose the single steps in case of an oxidative and reductive mechanism, respectively. These mechanistic points relate to electron transfer induced fragmentation to generate initiating radicals^[^ ^31^ ^]^ discussed much earlier considering the Hammond initiator comprising **MK** and benzophenone.^[^ ^27^ ^]^

(1) $$\begin{array}{c} \begin{array}{c} Sens^{*}+A^{+}X^{-}\to \left[\mathrm{Sen}s^{+•}+\left(Ar_{2}I^{•}X^{-}\right)\right]\to \mathrm{Sen}s^{+•}+A^{•}+X^{-}\\ ↓\\ \mathrm{Sens}+Ar_{2}I^{+}X^{-} \end{array} \end{array}$$

(2) $$\begin{array}{c} Sens^{*}\;+D-Y\to \left[\mathrm{Sen}s^{-•}+{\left(D-H\right)}^{+•}\right]\to \mathrm{Sen}s^{-•}+D^{•}+Y^{+}\\ ↓\\ \mathrm{Sens}+D-H \end{array}$$

Sens* reacts with either (**Ar_2_I** ^+^ **X** ^−^) or **D‐Y** (D: *p*‐Et‐O‐CO‐Ph‐N(CH_3_)‐) to form the respective species in the solvent cage disclosed in the Equation systems 1 and 2. Here, the decomposition of either (**Ar_2_I** ^•^ **X** ^−^) or **D‐H** ^+•^ must proceed faster as the electron back transfer proceeds resulting in the respective aryl radical **Ar** ^•^ and **D** ^•^, respectively. **C1 (Ar_2_I^+^X^−^)** functions as acceptor (**Ar_2_I** ^+^ **X** ^−^) in Equation 1 where the electron transfer yields the aryl radical (**Ar**•) after decomposition of the iodyl radical **Ar_2_I**•. Typically, **Ar_2_I** ^•^ fast decomposes though this transient has not been observed from our best knowledge. The reductive mechanism needs **C3** as donor to generate the respective **D** ^•^; that is *p*‐Et‐O‐CO‐Ph‐N(CH_3_)CH_2_•.

These mechanistic considerations require to determine the respective data to calculate the free energy of electron transfer Δ*G*_et_; that is the oxidation potential *E*_ox_, reduction potential *E*_red_, and excitation energy of the **Sens** *E*_exc_ to disclose the **PET** protocol from a thermodynamic point of view based on either Equations 1 or 2. Equation 3 shows the thermodynamic relationship to calculate Δ*G*_et_ (*F *= Faraday constant) to obtain a rough pattern about the thermodynamic scenario of the **PET**.^[^ ^32^ ^]^ Coulomb interactions were neglected in this expression. The excitation energy relates either to the energy of the participating exciting state; that is the S_1_ or the T_1_.

(3) $$\Delta G_{\mathrm{et}}=F\times \left(E_{\mathrm{ox}-}E_{\mathrm{red}}\right)-E_{\mathrm{exc}}$$

Table 1 summarizes the results obtained for **1**, **2a**‐**c**, and **3**. All Δ*G*_et_(red) data based on a reductive mechanism with **C3** (*E*_ox_ = 1.07 V^[^ ^81^ ^]^) as coinitiator, which is equivalent with Equation 2, resulted in a positive Δ*G*_et_(red), see Figures S1 and S2 for more information. The S_1_ was considered as source in this table. A consideration of the Δ*G*_et_ remaining between 0.12 to 0.42 eV address to the **PET** can proceed requesting a higher activation energy based on the Marcus theory^[^ ^82^ ^]^ resulting finally in a less efficient reaction.^[^ ^16^, ^18^ ^]^

**Table 1:** Summary of optical properties (*λ*_max_: absorption maximum; *ε*_max_: extinction coefficient at the maximum, *λ*_max_: fluorescence maximum, *Φ*_f_: fluorescence quantum yield, *E*_exc_: excitation energy of the singlet state), and electrochemical data related to oxidation (*E*_ox_) and reduction (*E*_red_) needed to calculation the free energy of electron transfer according to Equation 1 following an oxidative (Δ*G*_el_(ox)) or reductive mechanism (Δ*G*_el_(red)) for the sensitizers **1**, **2a‐c**, and **3**.

	Toluene					Acetonitrile						
**Sens**	*λ*_max_ (nm)^a)^	*ε*_max_ (M^−1^cm^−1^)^a)^	*λ*_f_ (nm)^a)^	*τ*_f_ (ps)^a)^	*Φ*_f_ (%)^a^)	*τ* _f_ (ps)	*λ* _f_ (nm)	*E*_ox_ ^a^ ^)^ (V)	*E*_red_ ^a^ ^)^ (V)	*E*_exc_ ^b^ ^)^ (eV)	Δ*G*_el_(ox)^c^ ^)^ (eV)	Δ*G*_el_(red)^d^ ^)^ (eV)
**1**	427	7500	490	337	2.8	479	515	0.40	−2.21	2.86	−1.77	0.42
**2a**	452	12 000	526	231	3.2	170	580	0.52	−1.98	2.60	−1.39	0.45
**2b**	396 443^e)^	15 700	505	291	1.8	459	570	0.42	−2.01	2.88	−1.77	0.20
**2c**	454	13 800	538	448	3.0	252	600	0.51	−1.94	2.65	−1.45	0.36
**3**	482	23 300	510		1.3	22	510	0.48^f)^	−1.57^f)^	2.52	−1.35	0.12

^a)^ Data measured in 0.1 M TBAPF_6_ /CH_3_CN, on Pt at 0.05 V s^−1^. Half‐wave potential (*E*_1/2_^0^)–for reversible process, peak potential (*E*_p_)–for an irreversible process.

^b)^ This relates to the onset of the S_1_, Supporting Information provide more details regarding determination of electrochemical data by taking the intersection point between absorption and fluorescence spectrum as a compromise to determine the excitation energy of the S_1_ (*E*_exc_).

^c)^ The *E*_red_ value of ‐0.68 V was taken for **C1** ^[^ ^85^ ^]^ to calculate Δ*G*_el_.

^d)^ The *E*_ox_ value of 1.07 V was taken for **C3** ^[^ ^81^ ^]^ to calculate Δ*G*_el_.

^e)^ Shoulder whose maximum was taken to to calculate Δ*G*_el_.

^f)^ Data were retaken to get a reliable set for comparison. Literature data^[^ ^80^ ^]^ used different reference electrode.

The scenario changes by consideration of an oxidative mechanism. Δ*G*_et_(ox) exhibits significant negative values with no remarkable difference for the thermodynamic situation of **PET** using **C1** as coinitiator, Table 1. Nevertheless, these considerations take the S_1_ as the excited state into account from where the **PET** would occur.

Stationary fluorescence data in acetonitrile and methanol exhibit a notable bathochromic shift of the emission take in acetonitrile. A higher dipole moment of the excited state explains this observation. Time resolved and stationary fluorescence spectroscopy additionally answers whether the S_1_ generates initiating radicals using **C1** as coinitiator. There was no dynamic quenching of the S_1_ as indicated by no change of the fluorescence decay time *τ*_f_ upon increase of the quencher concentration.

The fluorescence quantum yields *Φ*_f_ remain low. A strong nonradiative deactivation explains this scenario. Formation of the triplet state by intersystem crossing (ISC) of the ketones **1**, and **2a**‐**c** appears as the most likely pathway. Literature reported a quantum yield for ISC of about 0.99 of **MK**.^[^ ^54^ ^]^ Thus, the S_1_ should possess a minor function to generate initiating radicals. As a result, the T_1_ formed appears as the most likely transient contributing to the formation of initiating radicals.

**3** also follows this series regarding *Φ*_f_ but it does not form T_1_ vide infra. The rotation of the ylidene malononitrile moiety connects as an alternative to efficient non‐radiative deactivation. Similar structures operating as fluorescent probes in polymerization reactions support these findings.^[^ ^83^, ^84^ ^]^ No solvatochromic response existed in case of **3**.

### Transient Absorption Studies of the Triplet State

**Figure 2:**
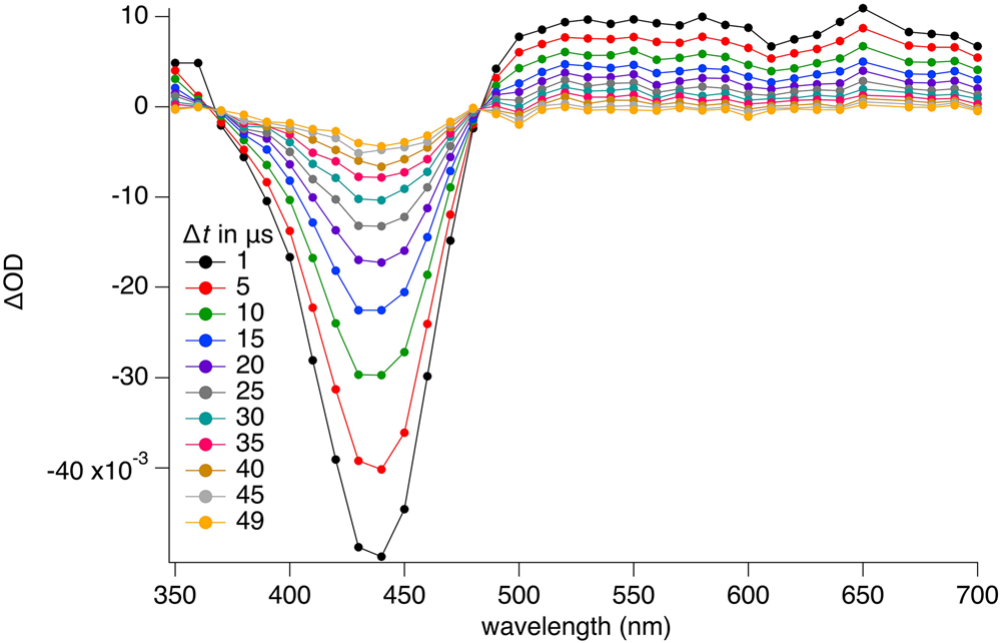
Transient absorption of **2a** in acetonitrile obtained with ns‐flash photolysis at different delay time Δ*t*, excitation wavelength: 355 nm.

Figure 2 exhibits the transient absorption (TA) spectra of **2a** using a standard setup with ns‐excitation.^[^ ^86^ ^]^ Figures S3–S5 provide more data regarding TA of **1**, **2b**, and **2c**. The negative OD‐values relate to ground state bleaching running through an isosbestic point at 464 nm from where the absorption of a transient starts covering a long wavelength. **3** did not show any TA in the microsecond frame. Thus, formation of T_1_ can be ruled out for this sensitizer.

Table 2 provides a summary of the data obtained. **1** and **2a**‐**c** form a transient with a lifetime in the microsecond region, which is comparable to that of **MK**.^[^ ^54^ ^]^ The transient in case of **1** and **2a‐c** relates to the triplet state. This was approved by triplet–triplet energy transfer experiments to 1,6‐dipheyl hexatriene (**DHT**, Scheme 2) serving as energy acceptor. Its low triplet state energy of 1.54 eV^[^ ^87^, ^88^ ^]^ enables triplet–triplet energy transfer from ^3^(**1**)* or ^3^(**2a‐c**)* to **DHT**. Quenching experiments resulted in a quenching constant in the order of 1.4…4.5 × 10^9^ M^−1 ^× s^−1^, see Table 2 for *k*_q_^T^(DHT). Alternative energy acceptors with higher triplet energy such as benzophenone (T_1_ energy: 2.97 eV in less polar solvent^[^ ^89^ ^]^) failed.

**Table 2:** Summary of photophysical constants and quenching constants with **C1**, **C2**, and **C3** of the triplet state (quenching constant *k*_q_^T^ obtained during transient absorption experiments (same conditions as in Figure 2, more details in Supporting Information).

**Sens**	*τ*_T_ (µs)	**C1** *k*_q_^T^ (M^−1^ s^−1^)	**C2** *k*_q_^T^ (M^−1^ s^−1^)	**C3** *k*_q_^T^ (M^−1^ s^−1^)	*E*_T_ ^a^ ^)^ (eV)	Δ*G*_el_^ox^(T) (eV)	Δ*G*_el_^red^(T) (eV)	*τ*_T_ (µs)	*k*_q_^T^(DHT) (M^−1^ s^−1^)
**1**	20.3	4.4 × 10^10^	2.30 × 10^10^	No reaction	2.39	−1.30	0.72	12.1	4.5 × 10^9^
**2a**	30.1	1.5 × 10^10^	1.89 × 10^10^	No reaction	2.25	−1.01	0.69	46.8	1.4 × 10^9^
**2b**	27.7	1.6 × 10^10^	3.03 × 10^10^	No reaction	2.45	−1.12	0.52	19.6	1.9 × 10^9^
**2c**	13.7	3.0 × 10^10^	2.50 × 10^9^	No reaction	2.40	−1.14	0.49	28.9	2.4 × 10^9^
**3**	No TA				No triplet			No TA	

^a)^ From phosphorescence measurements by extrapolation. TA: transient absorption.

Table 2 additionally shows T_1_ quenching experiments with **C1**, **C2**, and **C3**. As expected, no reaction proceeded with **C3** explainable by the positive Δ*G*_et_(red) reported in Table 1. On the other hand, diffusion‐controlled quenching occurred with **C1** and **C2** (*E*_red_ for **C2**: ‐1.24 V^[^ ^7^ ^]^). Here, the T_1_ successfully contributed the **PET** with onium salts **C1** and **C2** following an oxidative **PET** protocol of Equation 1. **C2** decomposes after **PET** into Ph• and Ph_2_S. Further experiments addressed to change the coinitiator because the reaction between ^3^(**2a**)* and **C1** resulted in a long living blue appearing transient which interfered data evaluation vide infra. **C2** resulted in a clearer optical pattern in the transient absorption experiment for data evaluation.

**Figure 3:**
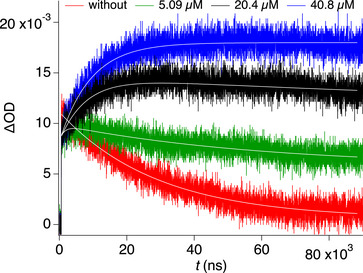
Kinetic traces **2a** obtained with different concentrations of **C2** in the transient absorption experiment. Same experimental conditions as in Figure 2.

Thus, flash photolysis provided additional data regarding the reactivity of ^3^ 2a* and **C2** in the **PET** protocol. Figure 3 shows the kinetic traces of **2a** obtained after adding different concentrations of **C2**. There exists a rise time with increasing **C2** concentration, which stands for the formation of the reaction product between ^3^ 2a* and **C2**; that is the cation radical **2a** ^+•^. The latter overlays at smaller **C2** concentrations. The exponential fit of the sample with the highest loading of **C2** (40.8 µM) resulted in a rise time of 9.2 µs, remaining in the time frame of the T_1_, and a species with much longer living species assigned to **2a** ^+•^ whose absorption fits in a similar spectral region as that of ^3^(**2a**)*. Spectroelectrochemical data shown in Figure 1 vide supra support these findings. Here, the cation radical exhibits a peak at 575 nm. The experiment with lower concentration of **C2** (5.09 µM) indicated a decay mostly dominated by remaining ^3^(**2a**)*.

A switching to **C3** as reactant showed no significant change of the kinetic traces because mainly the decay of ^3^ Sens* proceeded. It confirms again that **PET** based on either a reductive mechanism or H‐abstraction does not play a major role with these molecules confirming the discussion in Table 2 considering Δ*G*_el_.

### Singlet Oxygen and Mechanistic Consideration

The formation of the T_1_ requires consideration of ^1^O_2_ function. Equation 4 discloses its formation from ^3^ Sens*.

(4)

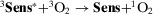




**Figure 4:**
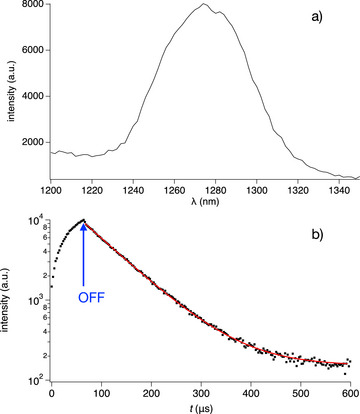
a) Emission spectrum of ^1^O_2_ generated by ^3^ 2a* as sensitizer in CD_3_CN. b)decay of ^1^O_2_ generated by ^3^ 2a* as sensitizer in CD_3_CN taken at 1280 nm.

Figure 4a exhibits the emission spectrum of ^1^O_2_ formed upon exposure of **2a** in air saturated CD_3_CN solution. The decay of ^1^O_2_, determined from data shown in Figure 4b, resulted in a decay time of 567 µs. This agrees with data generated with other sensitizers.^[^ ^90^ ^]^^1^O_2_ possesses strong oxidizing properties explaining the bleaching observed by exposure with the 395 nm emitting LED (Figure S8 illustrates the emission spectrum), see also Figures S9–S12 for the spectral changes of **1** and **2**.

**Figure 5:**
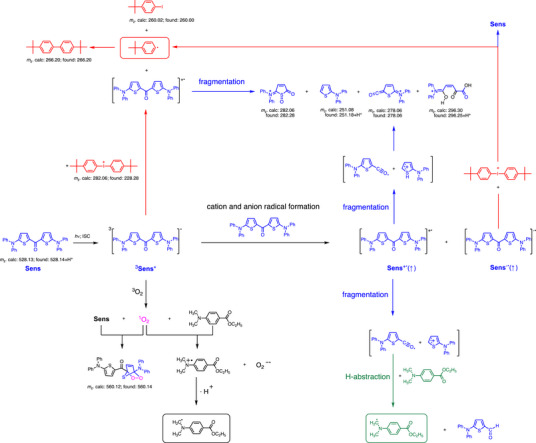
Mechanistic Proposal for the photoproducts formed for exposure of **2a** in the presence of **C1** or **C3** based on mass spectrometric study. It also shows the molecular ions observed obtained after irradiation of **2a** with **C1** using a UV LED emitting at 470 nm (Kulzer) with an intensity of 1 W cm^−2^ (0.1 cm distance from the sample). Exposure was pursued at the absorption edge because concentration was higher needed for the mass spectrometric experiment to facilitate deeper penetration of light in the sample.

The availability of photogenerated, non‐inhibiting ^1^O_2_ requires to include this reactant in the mechanistic discussion. Cycloaddition reactions and oxidation reactions present some examples disclosing its consumption and conversion of ^3^O_2_ into non‐inhibiting species. The mechanistic proposal shown in Figure 5 outlines main pathways contributing to initiating radicals. Different competitive pathways occur upon exposure of **2a** after addition of either **C1** or **C3**. This focusses for reactions proceeding from the T_1_ because fluorescence experiments approved negligible reactivity of the S_1_ as shown in Table 2 vide supra. Absorption decreased upon exposure of **2a** with **C1**, see Figure S13. This proceeded much faster compared to Figure S10.

Figure 5 illustrates a proposal of the structures formed based on the molecular ion peak found in mass spectrometry in case of the reaction between **2a** and **C1**. Mass spectrometry identified the masses at 266.20 and 260.02 *m*_z_ for (*p*‐C_4_H_9_‐Ph)_2_ and the iodoarene *p*‐C_4_H_9_‐Ph‐I, respectively, originating from the cation of **C1**. **PET** from ^3^ **2a** ^*^ to **C1** yields the respective iodyl radical. It immediately decomposes ultrafast to *p*‐C_4_H_9_‐Ph‐I and *p*‐C_4_H_9_‐Ph•. The latter recombines to (*p*‐C_4_H_9_‐Ph)_2_.

The identified oxidation products of **2a**, shown in blue color, can be formed by cleavage of the thienyl ─C═O bond of **2a** ^+•^. This relates to the molecular ions of 279.06 *m*_z_, 229.28 *m*_z_, and 251.18+H^+^ *m*_z_. The distorted structure of of **2a** ^+•^ can favor this pathway while cyclization to a planar pattern does not appear as an option. This is concluded from investigations using a similar cation radical of an aromatic ketone; that is **MK**,^+•[^ ^80^ ^]^ and complementary mass spectrometric studies of **MK**s.^[^ ^91^ ^]^ It seems most likely that **2a** follows similar pathway as **MK** regarding the cleavage of **2a** ^+•^. The cation formed in the cleavage can also stabilize by further oxidation and loss of sulfur as shown for the ion 296.25+H^+^ *m*_z_. Loss of sulfur in thiophenes does not appear as unusual as shown for alternative systems of thiophene based drugs.^[^ ^70^ ^]^

The ^1^O_2_ formed can add to the thienyl moiety forming the [4+2] cycloaddition product with *m*_z_ of 560.14. Previous reports of thiophenes support this finding.^[^ ^77^ ^]^ Remarkably, all thienyl ketones exhibit after exposure in the mass spectrum an additional signal showing an increase of 32 *m*_z_ with respect to the molecular ion of **1** or **2a**‐**c** (see Figures S6 and S7 for **2a**). Thus, the T_1_ of the **TK**s converts inhibiting ^3^O_2_ to non‐inhibiting ^1^O_2_. It can react in a [4+2] cycloaddition reaction with the thiophene moiety on the one hand side. On the other hand, ^1^O_2_ can oxidize tertiary amines such as **C3** resulting in formation of initiating amino alkyl radicals.^[^ ^92^ ^]^ This additionally explains the formation of polymer network observed in practical systems under air. The superoxide radical formed does not contribute to inhibition of radical polymerization.^[^ ^61^ ^]^

The explanations vide supra apply to an oxidative mechanism where Δ*G*_el_^ox^(T) between 2a* and **C1** is ‐1.01 eV indicating strong exergonic character. Notably again, the triplet state does not react with **C3**, see Table 2, and therefore actually excludes a **PET** between them. The reaction between 2a* and **C3** results in an endergonic scenario with Δ*G*_el_^red^(T) = 0.69 eV. However, the experiments on photopolymerization vide infra showed also initiation with **C3** when working with **1** or **2** as sensitizer. A deeper understanding for initiation requires to consider the reaction between two molecules of **2a** where one remains in the T_1_ while the other is in its ground state. This reaction forms a radical cation and radical anion. Their recombination forms according to the spin statistics the T_1_ in 75% yield while the remaining part assigns to the S_1_. The formation of these both radical ions would result in a slightly endergonic Δ*G*_el_(T) of 0.25 eV and may explain the slower polymerization rate shown vide infra. Such reactions can occur under endergonic conditions under some circumstances.^[^ ^93^ ^]^

Electrochemical investigations vide supra discuss a much better stability of **2a** ^−•^, which can react with **C1** to give the respective initiating aryl radical. The Δ*G*_el_ between **2a** ^−•^ and **C1** appears clearly negative (‐1.3 eV) supporting this protocol from a thermodynamic point of view. On the hand, the insufficient stability of **2a** ^+•^, shown in Figure 1 by electrochemical experiments, favors its decomposition into the radical and cation intermediates shown. The thienyl ─C≡O^•^ radical favors H‐abstraction from **C3** resulting in the initiating aminyl radical.

Formation of **2a** ^+•^ and **2a** ^−•^ can proceed in general no matter which coinitiator was added. Such reactions do not appear as unlikely and were disclosed for charge‐separated donor–acceptor sensitizers systems showing thermally activated delayed fluorescence (TADF).^[^ ^94^ ^]^ This gave a deeper pattern regarding the mechanism.

### Radical Photopolymerization Under Air and Nitrogen

**Figure 6:**
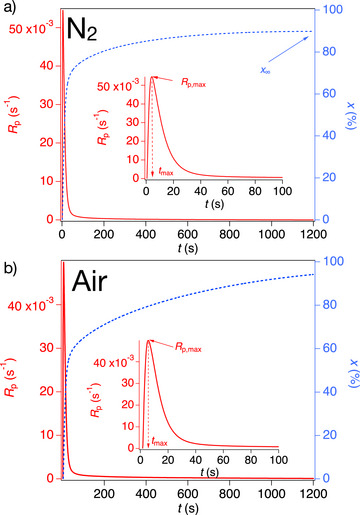
Photo‐DSC of **2a** with the coinitiator **C1** in the monomer mixture TPGDA:UDMA = 2:3 under aerobic and anaerobic conditions applying UV‐LED as exposure source emitting at 395 nm with an intensity of 195 mW cm^−2^. Experimental setup according to ref.[50, 66].

The sensitizers **1**, **2a**‐**c**, and **3** were investigated in combination with either **C1** or **C3** for photoinitiation of the monomer mixture **UDMA**:**TPGDA** = 3:2 (see Scheme 1 for structures). **1**, and **2a**‐**c** initiated conventional radical photopolymerization even under air, which can be seen as great feature. A photo‐DSC setup^[^ ^50^ ^]^ operating at 395 nm LED exposure enabled to pursue experiments under aerobic and anerobic conditions. Figure 6 shows the data obtained under nitrogen and air. The polymerization rate *R*_p_ at its maximum (*R*_p,max_) slightly decreases by switching the environment from nitrogen (Figure 6a) to air (Figure 6b). Typically, other multi‐component photoinitiating systems comprising *i*‐propyl‐thioxanthone do not respond under air. Comparison of the final conversion (*x*_∞_) obtained in both environments shows only a modest drop under air.

A detailed comparison of the kinetic traces in Figure 6a,b (Tables S2 and S3) exhibits a slower increase of the conversion after passing *R*_p,max_. The mechanism shown in Figure 5 explains these findings. The thienyl ketones **1** and **2a**‐**c** successfully form singlet oxygen (Figure 4). This adds to the thiophene moiety in a [4+2] cycloaddition resulting in consumption of the same being part of the sensitizer. Thus, the cycloaddition of ^1^O_2_ to the thiophene moiety connects to a decrease of sensitizer needed to generate initiating radicals. The conversion of inhibiting ^3^O_2_ to non‐inhibiting ^1^O_2_ represents undoubtedly one possible reason to explain the oxygen tolerance found. It may explain the slower increase in conversion after passing *R*_p,max_.

Table 3 summarizes the reactivity data obtained under nitrogen and air. Samples with **2**, comprising a diaryl amino moiety, exhibit a higher reactivity compared to **1** bearing the morpholino group as amino function, as concluded from the *R*_p,max_ and *x*_∞_ data. Here, **2a** and **2c** possess the most robust tolerance in air surrounding during photopolymerization with **C1**.

**Table 3:** Summary of photo‐DSC data of the different sensitizers of **1**–**3** with the coinitiator **C1** or **C3** in the monomer mixture TPGDA:UDMA = 2:3 under aerobic and anaerobic conditions applying UV‐LED as exposure source emitting at 395 nm with an intensity of 195 mW cm^−2^. Experimental setup according ref.[50, 66].

		Nitrogen			Air		
Sens	Coinitiator	*R*_p,max_ (s^−1^)	*t*_max_ (s)	*x*_∞_	*R*_p,max_ (s^−1^)	*t*_max_ (s)	*x*_∞_
**1**	**C1**	3.15 × 10^−2^	10	76	7.8 × 10^−3^	17	56
	**C3**	3.37 × 10^−4^	23	10	1.8 × 10^−4^	13	10
**2a**	**C1**	5.5 × 10^−2^	4	90	4.9 × 10^−2^	6	94
	**C3**	7.24 × 10^−3^	34	98	4.5 × 10^−3^	38	80
**2b**	**C1**	6.0 × 10^−2^	4	96	3.2 × 10^−3^	5	87
	**C3**	3.88 × 10^−3^	18	93	1.9 × 10^−3^	25	69
**2c**	**C1**	4.6 × 10^−2^	4	91	4.2 × 10^−2^	7	88
	**C3**	5.3 × 10^−3^	43	86	3.7 × 10^−3^	54	75
**3**	**C1**	1.2 × 10^−2^	11	77	No reaction		
	**C3**	5.8 × 10^−4^	15	7	No reaction		

Switching to **C3** as coinitiator did not change the scenario in general in case of **2a**‐**c**, Table 3. **2a** and **2c** showed the best tolerance under air. **1** exhibited less reactivity compared to **2** considering the system under air and nitrogen. Thus, introduction of the diarylamino pattern in the thienyl ketone moiety responsibly increases the reactivity. Again, transient absorption approved no reaction between the triplet state of **1** or **2** with **C3**. These findings, support the mechanistic scheme in Figure 5.

The conversion of inhibiting ^3^O_2_ to non‐inhibiting ^1^O_2_ by the T_1_ could be seen as one reason to discuss the air tolerance during photopolymerization. It can be consumed in a [4+2] cycloaddition and/or react with amines resulting in the O_2_^−•^ radical and the respective cation radical of the amine. The latter forms the initiating aryl(amino)alkyl radical.^[^ ^81^, ^95^ ^]^ Anaerobic conditions require formation of a well reducible species. The cation radical of the thienyl ketone can function as precursor to yield radicals after its fast decomposition that react with the amine to give the initiating radical, Figure 5.

**3** only worked under anaerobic conditions in an oxidative mechanism achieving acceptable reactivity. No crosslinking occurred by switching to a reductive mechanism with **C3** while similarly to **1** only small amount on polymer was formed. This sensitizer showed no triplet formation. Thus, the S_1_ serves as source in the **PET** to generate initiating radicals. Here, the rotation of the [dithienylidene]malodinirile moiety in the excited state can be seen as an efficient non‐radiative deactivation channel resulting additionally in a drop of photoinitiation efficiency for radical polymerization. Similar patterns worked well with [benzylidene]malodinitriles to probe free volume in polymers.^[^ ^83^, ^84^ ^]^ Here, the rotational efficiency decreased with increased viscosity, which may explain a certain reactivity reported for the oxidative mechanism.

These results enable to conclude that efficient triplet formation can be seen as a prerequisite to obtain a good photoinitiation efficiency. Furthermore, the diarylamino pattern with the proposed formation appears more favorable to form the respective radical cation and radical anion of the thienyl ketone. This seems to proceed with less importance in **1** and **3** explaining the lower reactivity.

**2a** mostly can be seen as an attractive candidate from a practical point of view. This also includes consideration of the reactivity in radical photopolymerization. For further exploration, dynamic mechanical analysis (DMA) presents an attractive tool to examine the glass transition based on storage (*E*′) and loss modulus (*E*″). **2a** was investigated either with **C1** or **C3** in the monomer mixture of **UDMA** and **TPGDA**. The data obtained in this study indicated a higher *T*_g_ in the case of reaction with **C1** (comparing the data available at *T*_g_(*E*′), *T*_g_(*E*″), and *T*_g_(tan δ) while initiation with **C3** as coinitiator gave significant lower values, Table 4. Longer chains between the crosslink junctions can be seen as one parameter causing a decrease of *T*_g_. The average molecular weight between the crosslinking points (*M*_c_) represents one quantity explaining this observation. Determination of *E*″ in the viscoelastic plateau; that is 150 °C in this example (Figure S14), gives access to this quantity, Equation 5.

(5) $$M_{c}=\frac{3\mathrm{RT}}{E^{\prime }}\;\times \rho$$

**Table 4:** Summary of the network data made by **2a** and the coinitiator **C1** or **C3** in the monomer mixture **TPGDA**:**UDMA** = 2:3 under aerobic conditions (see Supporting Information for more information regarding sample preparation) applying UV‐LED exposure at 395 nm as exposure source emitting with an intensity of 1 W cm^−2^. Experimental setup according to a previously disclosed setup for DMA.^[^ ^66^ ^]^ Intensity LED and sample was adjusted by change of the distance between them resulting in 3 cm here.

	*M*_c_ (g mol^−1^)	*T*_g_(*E*′) (°C)	*T*_g_(*E*″) (°C)	*T*_g_(tanδ) (°C)	*E*′(*T*_g_) (GPa)	*E*″ (*T*_g_) (GPa)	tanδ
**C1**	238	0	49	82	2.6	0.16	0.36
**C3**	380	−31	7	56	2.9	0.17	0.38

(ρ = density of the material, *T* = temperature, *R* = gas constant, storage modulus at the temperature *T* in the viscoelastic plateau)

Data evaluated from the graph resulted for the monomer mixture of **TPGDA** and **UDMA** in a *M*_c_ of 238 and 380 g mol^−1^ in case of the system **2a**/**C1** and **2a**/**C3**, respectively. Thus, networks made by reaction with **C1** gave more densely crosslinked materials compared to those made with **C3**, Table 4. It confirms findings discussed based on *T*_g_ vide supra. The different reactivity, which was less in case of **C3** as coinitiator, may explain the distinct network density obtained.

Mechanical parameters of the networks, such as *E*′ and *E*″ at the respective *T*_g_, exhibited similar data using either **2a**/**C1** or **2a**/**C3** as photoinitiation system, Table 4. Thus, different initiation kinetics to form the network either with **2a**/**C1** or **2a**/**C3** did not interfere the mechanical properties while differences exist regarding *T*_g_. Obviously, the higher final conversion did not have the expected impact on *T*_g_. This may be explained by the formation of crosslinked structures occurring much earlier, while the final stage of polymerization does not significantly contribute to this material parameter.

### Cytotoxic Studies

**Figure 7:**
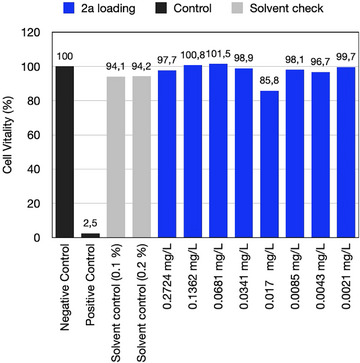
Mean value and standard deviation (*n* = 4) of **CHO‐9** cell vitality upon addition of **2a** with different loadings in DMSO/water as processing medium. In addition, control samples (positive, negative) and the maximum DMSO tolerance for **CHO‐9** cells are specified before cytotoxic effects (cell vitality <80%) occurred. To rule out cytotoxic effects of the solvent, the sample solution is diluted by a factor of 1000. This results in a maximum test concentration of 0.1 wt%. To achieve even higher test concentrations, an additional dilution factor of 500 was used resulting in maximum test concentrations of 0.2 wt%.

These promising results brought **2a** to cytotoxic studies to get a first rough pattern regarding possible toxicological issues. Thus, several concentrations were tested for their potential to inhibit cell viability of **CHO‐9** cells in a MTT assay. Figure 7 shows the mean values with standard deviation from four separate replicate experiments. The sample, dissolved in DMSO (136.22 g L^−1^), was diluted ruling out the possibility that the solvent DMSO can cause the cytotoxic effects. This was required because **2a** itself possesses a poor compatibility with aqueous surrounding. The test included two solvent controls which were carried with and without DMSO receiving an answer on negative and positive control, respectively. Thus, the sample comprising different DMSO loadings obtained after dilution showed no significant response. This leads to the conclusion that **2a** can be seen as low cytotoxic under the experimental conditions chosen. It presents a big feature to use this compound as **Sens** in multi‐component photoinitiating systems. These results also address the challenge to pursue explorative studies of alternative heterocyclic structures if they would give a high cell vitality either. These results open the possibility to use these sensitizers as alternatives for those where toxicological issues appeared.^[^ ^37^, ^38^, ^39^, ^55^, ^69^ ^]^ A combination with **C1** would be practical because this coinitiator also exhibits low cytotoxicity.^[^ ^50^ ^]^

## Conclusion

The T_1_ of the **TK**s possesses a key function to form intermediates for initiation of radical polymerization. A comprehensive exploration of the excited state by fluorescence and transient absorption spectroscopy, electrochemical studies with focus on cyclic voltammetry and spectroelectrochemistry enabled access to mechanistic aspects of the **PET** responsibly generating initiating radicals. Photo‐DSC studies under anaerobic and aerobic conditions gave convincing results to prove that radical polymerization can efficiently proceed also under air. DMA experiments approved the formation of polymer network either to an oxidative or reductive mechanism.

The exceptionally good oxygen tolerance of systems comprising of diarylamino(dithienyl) ketones represents a feature that opens new options for practical applications. The T_1_ possesses a key function to generate initiating species with **C1** or the amine **C3** under both aerobic and anaerobic conditions. While ^1^O_2_ uptakes a key function to explain the good performance also under air, intermediates formed can additionally lead to initiating radicals by H‐abstraction of intermediates formed in a complex mechanism between several sensitizer molecules. This can bring additional impact regarding the design of multi‐component photoinitiating systems in the future.

The low cytotoxic response showing alternatives to current sensitizers in multi‐component systems comprising **ITX**, known for its issues in food packing coatings, represents an additional feature. It could be additionally seen as an alternative of one‐component photoinitiating systems where the cytotoxicity of TPO demands to develop alternatives. Particularly, the combination of **2a** with either **C1** or **C3** represents an interesting alternative for future design strategies to design efficient photoinitiating systems with low cytotoxicity. Future work may also design patterns of **C3** where the carboxylic acid group is esterified with polyols resulting in multi‐functional radical initiator comprising many reactive groups for H‐abstraction resulting in initiation of radical polymerization. This must impact network formation.

Furthermore, the question still arises whether thiophene moieties would be necessary to obtain the necessary performance. Future studies shall focus on alternative heterocycles based on furane or pyrrole moieties keeping available the diene pattern of the heterocycle for the 4+2 cycloaddition with singlet oxygen. Though, one keeps in mind the role of the T_1_ as a prerequisite.

## Supporting Information

Supporting information provides more information regarding the materials used for the studies. It additionally describes experimental conditions to pursue absorption spectra, fluorescence measurements, singlet oxygen detection, spectroelectrochemical data acquisition and CV, transient absorption spectra, exposure experiments, mass spectrometric analysis of the photoproducts, photo‐DSC experiments, and polymer network characterization with DMA.

## Conflict of Interests

The authors declare no conflict of interest.

## Supporting information



Supporting Information

## Data Availability

The data that support the findings of this study are available from the corresponding author upon reasonable request.
