# Supplementary material for: Bis(2‐amino‐5‐thienyl)Ketone as Oxygen Tolerant Sensitizer for Conventional Radical Photopolymerization
Source: Angew Chem Int Ed Engl. 2025 Dec 16;65(5):e18608. doi: 10.1002/anie.202518608 (PMC12851004; doi:10.1002/anie.202518608)
Supplement: Supplementary file 1 — Supporting Information [file ANIE-65-e18608-s001.docx]

[1. Materials 2](#_Toc216533493)

[2. Electrochemical Investigations 2](#_Toc216533494)

[2.1. Electrochemical measurements 2](#_Toc216533495)

[2.2. EPR/UV−Vis−NIR spectroelectrochemical measurements 3](#_Toc216533496)

[3. Characterization by spectroscopic methods 5](#_Toc216533497)

[3.1. UV-Vis spectroscopy 5](#_Toc216533498)

[3.2. Time-Resolved Fluorescence and Steady State Spectrocopy 5](#_Toc216533499)

[3.3. Fluorescence quantum yields 5](#_Toc216533500)

[3.4. Spectroscopic detection of singlet oxygen 6](#_Toc216533501)

[3.5. Transient absorption spectroscopy 6](#_Toc216533502)

[4. Mass-Spectrometric Investigations 8](#_Toc216533503)

[5. Light sources and exposure experiments 9](#_Toc216533504)

[5.1. Light sources 9](#_Toc216533505)

[5.2. Exposure 9](#_Toc216533506)

[6. Thermoanalytical methods 11](#_Toc216533507)

[6.1. photo-DSC 11](#_Toc216533508)

[6.2. DMA 14](#_Toc216533509)

[7. Cytotoxic studies 14](#_Toc216533510)

[7. ROHS Statement 26](#_Toc216533511)

[8. References 27](#_Toc216533512)

# 1. Materials

The monomers tri(propylene glycol) diacrylate (**TPGDA**) and urethane dimethacrylate (**UDMA**) were purchased from Merck (Sigma-Aldrich). **TPGDA** was run through basic Al_2_O_3_ (Carl Roth GmbH) to remove the inhibitor before used for polymerization, while all the other monomers were taken without any further purification. Solvents used were spectroscopic grade and purchased from Merck (Sigma-Aldrich) either, which also served as supplier for deuterated solvents. Synthesis of the sensitizers **1**-**3** was previously disclosed[S1-4]. The coinitiator **C1** was purchased as S 2430 from FEW Chemical GmbH, Germany. The sulfonium salt **C2**, the amine **C3**, 1,6-dihenylhexatriene (DHT), and Michlers Ketone (**MK**) were obtained from Merck (Sigma-Aldrich).

# 2. Electrochemical Investigations

## 2.1. Electrochemical measurements

Cyclic voltammetry (CV) was carried out on a PARSTAT4000 potentiostat (Princeton Applied Research, Ametek, Germany) in a three-electrode cell in degassed anhydrous acetonitrile solution containing 0.1 M of tetra-*n*-butylammonium hexafluorophosphate (TBAPF_6_) as a supporting electrolyte. A Pt disk, AgCl-coated silver wire, and Pt sheet electrode were used as the working electrode, the reference electrode, and the counter electrode, respectively. The Pt electrodes were polished with 1 μm diamond and 0.3 μm alumina suspension, sonicated, rinsed in double distilled water, and then air-dried. All potentials in this work are given versus Fc/Fc^+^ redox couple as an internal standard. The electrochemical measurements were performed under nitrogen atmosphere and at ambient temperature.

Figure S1. Cyclic voltammogram of **TK**s and **MK** measured on Pt in acetonitrile solution containing 0.1 M TBAPF_6_ at a scan rate of 0.05 V/s.

## 2.2. EPR/UV−Vis−NIR spectroelectrochemical measurements

For EPR measurements, EMX X-band CW spectrometer (Bruker, Germany) at 100 kHz modulation was used. The spectra were recorded in an optical EPR cavity allowing the connection of two optical wave guides to measure the electronic absorption spectra *in situ* in transmission mode and the EPR spectra simultaneously. An NMR teslameter was used for precise *g* value determination. The UV-Vis-NIR spectra were measured using the Avantes spectrometer AvaSpec-2048×14-USB2 with the CCD detector and AvaSpec-NIR256-2.2 with the InGaAs detector (Avantes, The Netherlands). A light source Avantes Avalight-DH-S-BAL was used. Both, the EPR spectrometer and the UV−Vis−NIR spectrometer are linked to a HEKA potentiostat PG 390 which triggers both spectrometers. At ambient temperature, the EPR and UV−Vis−NIR spectra were collected at a continuous potential scan rate (ca. 5 mV/s). Each UV−Vis−NIR spectrum was collected relative to that of the neutral (uncharged) compound. In spectroelectrochemical experiments, an EPR flat cell with a laminated gold μ-mesh (Goodfellow, UK) as working electrode, an AgCl-coated silver wire as reference electrode, and a platinum wire as counter electrode was used. The cell assembling was done under nitrogen atmosphere.

|  |  |
| --- | --- |
|  |  |

Figure S2. In situ UV–Vis–NIR absorption spectra of **1** (a), **2b** (b)**, 2c** (c) and **3** (d) measured during the reduction process. Inset: EPR spectrum of corresponding radical anions.

Table S1. EPR and UV–vis–NIR spectroscopic data for electrochemically generated radical anions.

| compound | *hfc* constants, Gauss  (number of nuclei) | | | | | *g* value | absorption bands (λ_max_), nm |
| --- | --- | --- | --- | --- | --- | --- | --- |
|  | *a*(^1^H_β1_) | *a*(^1^H_β2_) | | *a*(^14^N_amine_) | *a*(^14^N_CN_) |  |  |
| ^1^**1^•−^** | - | - | - | | - | - | 455, 767 |
| **2a^•−^** | 4.33 (2H) | 0.99 (2H) | 0.61 (2N) | | - | 2.0044 | 494, 802 |
| **2b^•−^** | 4.33 (2H) | 0.98 (2H) | 0.57 (2N) | | - | 2.0044 | 487, 825 |
| **2c^•−^** | 4.31 (2H) | 0.99 (2H) | 0.62 (2N) | | - | 2.0044 | 500, 813 |
| **3^•−^** | 4.84 (2H) | 1.62 (2H) | 0.54 (2N) | | 1.08 (2N) | 2.0029 | 430, 701 |

^1^ analysis of the EPR signal was not possible due to the overlapping with signal from the electrolyte solution generated at high negative potential.

# 3. Characterization by spectroscopic methods

## 3.1. UV-Vis spectroscopy

UV-Vis spectra were recorded using a UV-3600i Plus in 1×1 cm quartz cuvettes using the solvent as reference.

## 3.2. Time-Resolved Fluorescence and Steady State Spectrocopy

The regular fluorescence set up (FluoTime 300 from Picoquant GmbH) was carried for recording fluorescence emission λ^f^_max_, to take decay time τ_f_, and quantum yield Φ_f_ with the excitation from a diode laser (LDH-P-C-375; Picoquant GmbH Germany) emitting at 376 nm. Time correlated single photon counting was the technique to take the emission decay. This instrument from Picoquant GmbH provides the opportunity to measure decay between 25 ps and several milliseconds. The instrument was equipped with high resolution emission double monochromator operated in subtractive mode (2x 300 mm focal length, UV/VIS/IR grating pair 1st stage: 600 l / mm blaze 1250 nm and 1200 l / mm blaze 500 nm, dispersion: 4.5 nm / mm, UV / VIS grid pair 2nd stage: 1200 l / mm blaze 500 nm 1200 l / mm blaze 500 nm (-), 2.7 nm / mm (-), PMA-C 192-M Cooled photomultiplier) for the emission side. Excitation proceeded by using a computer-controlled diode laser driver for picosecond pulses (196 kHz and 80 MHz, external trigger input). The system can be also operated in *cw*-mode to record spectra in additive mode (monochromator: blaze 500 nm (+), dispersion: 1,4 (+)). The diode laser (LDH-P-C-375; Picoquant GmbH Germany) used operated at 376 nm. EasyTau software and laser control software controlled the system. This also facilitated to calculate decay time by the software by iterative convolution. An appropriate scatter (Ludox in water) was taken to record the instrumental response function of the system. Time resolution of the instrument is >25 ps. For preparation, the sample was dissolved in methanol (spectroscopic grade) to get the absorption of extinction of about 0.1 at 376 nm. The EasyTau Software also operated to calculate the decay time by iterative convolution between the instrumental response and the sample.

## 3.3. Fluorescence quantum yields

Absolute fluorescence quantum yields were measured with an integrating sphere provided by Picoquant GmbH for the FluoTime 300 spectrometer. First the respective counts available by the laser were measured using a blanc cuvette comprising spectroscopic grade solvent. Then, the sample was changed with a cuvette comprising the same solvent and the absorber exhibiting an absorption of about 0.1 at 376 nm. This treatment resulted in the absorption of the absorber taken for measurement needed to determine fluorescence quantum calculated with the EasyTau software that also controlled the instrument.

## 3.4. Spectroscopic detection of singlet oxygen

Singlet oxygen detection was pursued with the same configuration disclosed *vide supra* with an extended red-sensitive PMT from Picoquant GmbH. This provides the detection of ^1^O_2_ emission between 950-1400 nm.

Steady state spectra.

Singlet oxygen emission was recorded between 1200-1350 nm applying a diode laser (LDH-P-C-375; Picoquant GmbH Germany) excitation at 376 nm. Optical density at the excitation wavelength was about 0.2. EasyTau software from Picoquant GmbH operated to control the experiment. Experiments were pursued in deuterated solvent (CD_3_CN) where singlet oxygen exhibits a notably higher lifetime.

Lifetime determination.

The burst mode excitation was applied to take the lifetime of ^1^O_2_. The software EasyTau optimized the burst length and number of excitation pulses to receive an acceptable signal based on the instrument sensitivity. . Experiments were pursued in deuterated solvent (CD_3_CN).

## 3.5. Transient absorption spectroscopy

Laser flash photolysis measurements were carried out using a LP980 (Edinburgh Instruments) equipped with a 150 W ozone-free flash lamp, a R928 photomultiplier (Hamamatsu), a Czerny-Turner monochromator and a digital oscilloscope (Tektronix). Excitation pulses were produced by a Surelite Nd:YAG laser (Continuum) working at 10 Hz and pumping an optical parametric oscillator (Horizon, Continuum) which can deliver nanosecond pulses between 225 nm to 1800 nm. This system was used to pump the sample at 355 nm with 7 mJ/pulse using a collinear setup. All samples exhibited an absorbance of about 0.5 at the excitation wavelength and were bubbled with Ar prior to the experiment.

Figure S3. Transient absorption of **1** in CH_3_CN

Figure S4. Transient absorption of **2b** in CH_3_CN

Figure S5. Transient absorption of **2c** in CH_3_CN

# 4. Mass-Spectrometric Investigations

Mass spectrometry identified the mass of the products disclosed in the synthetic procedure and exposure experiemnts. The mass spectrometer was coupled with a HPLC system (Vanquish MD HPLC von ThermoFisher Scientific) operated with a mobile phase of CH_3_CN:H_2_O=80:20 (column Hypersil C4 (125 x 4mm) from Thermofischer Scientific). The mass spectrometer operated in positive mode (Elektrospray-Ionisation; ESI). A Orbitrap IQ-X Tripid-Mass detector from ThermoFisher Scientific detected the ions.

Figure S1. Mass spectrum of 2a dissolved in CH_3_CN.

Figure S7. Mass spectrum of 2a dissolved in CH_3_CN after exposure at 470 nm for 20 s with a proposal of the [4+2] cycloaddition product. Figure S8 shows the emission of the LED *vide infra*.

# 5. Light sources and exposure experiments

## 5.1. Light sources

Different LEDs operated to determine the photochemical reactivity of **1**-**3**. These light sources originated from Lumitronix (395 nm) und Kulzer (470 nm). Figure S8 shows the respective emission spectra. Light intensity was adjusted by changing the distance between sample and LED. This resulted in about of 1 cm taking a fiber optical spectrometer (USB4000 from Ocean Optics) as radiometer sung the 395 nm LED from Lumitronix while the distance between 470 nm LED and sample was about 0.1 cm.


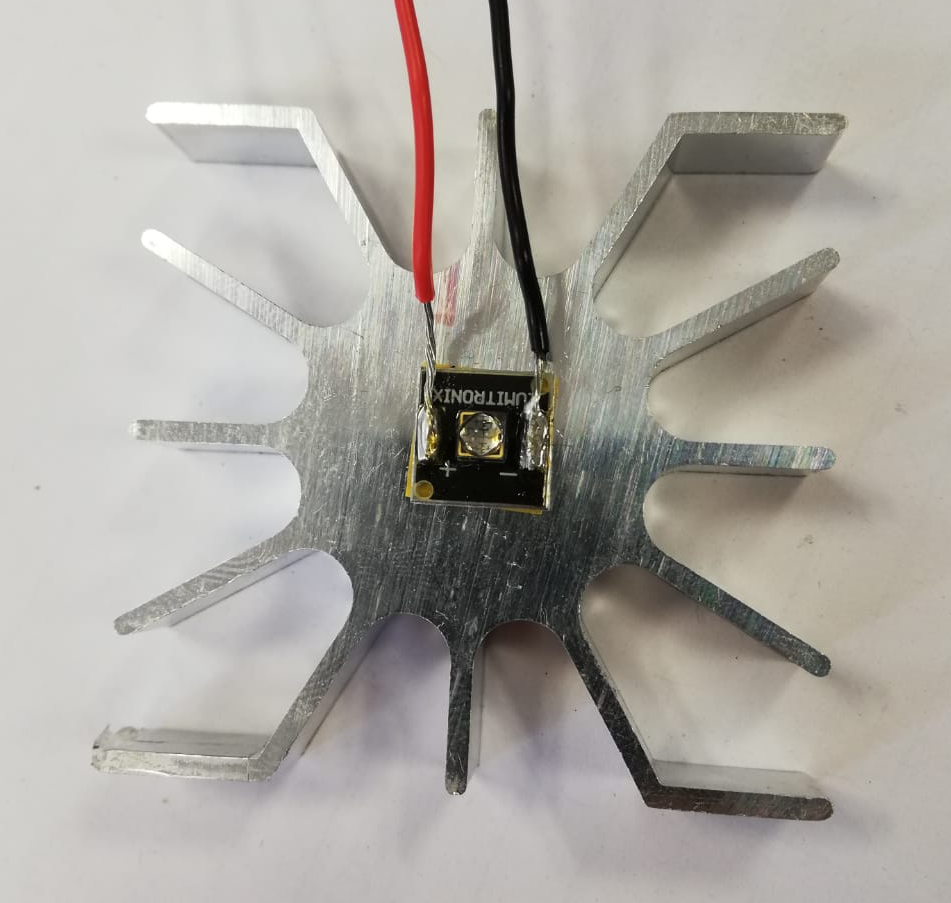


Figure S8. Emission spectra of the 395 nm LED and 405 nm LED (Lumitronix), and that of Kulzer operating at 470 nm LED. The photograph shows the mounted LED (Lumitronix) on a cooling block.

## 5.2. Exposure

The sensitizers were dissolved in CH_3_CN at a concentration of 10^-5^ M. The exposure wavelength for UV-Vis kinetics was 395 nm operating with an intensity of 175 mJ/cm^2^. LED exposure was pursued in 1×1 cm cuvette in 5 s time intervals under continuous stirring. The exposed solution was stirred for additional 2 min in the dark before the spectrum was taken. Figures S9-12 show the spectra obtained for the sensitizers **1**, **2a**, **2b**, and **2c**, respectively, upon exposure under air without **C1**. Figure 13 depicts the bleaching of **2a** in the presence of **C1**.

Figure S9. UV-Vis spectra of **1** (c=21.8 μM) obtained upon exposure under air without **C1** in acetonitrile. Time interval between each exposure cycle: 5s.

Figure S10. UV-Vis spectra of **2a** (c=16.3 μM) obtained upon exposure under air without **C1** in acetonitrile. Time interval between each exposure cycle: 5s.

Figure S11. UV-Vis spectra of **2b** (c=11.3 μM) obtained upon exposure under air without **C1** in acetonitrile. Time interval between each exposure cycle: 5s.

Figure S12. UV-Vis spectra of **2c** (c=13.7 μM) obtained upon exposure under air without **C1** in acetonitrile. Time interval between each exposure cycle: 5s.

Figure S13. UV-Vis spectra of **2a** (c=16.3 μM) obtained upon exposure under air with **C1** (c=19.3 μM) in acetonitrile. Time interval between each exposure cycle: 5s.

# 6. Thermoanalytical methods

## 6.1. photo-DSC

A standard photo-DSC setup[S5] was used to determine the photoinitiation efficiency of the obtained **CD-PI**s in the monomer mixture of **UDMA** and **TPGDA** using a 365 nm LED with an emission intensity of 200 mW/cm^2^. The sample for the photo-DSC experiment was the same solution used for real-time FTIR measurements with a sample size of around 5 mg. The investigation was carried out at 25°C cooled by liquid nitrogen under nitrogen and airflow, respectively. The generated light was collected by a lens and projected into a y-fiber connected to the head of the DSC (Q2000 from TA-Instruments), resulting in similar intensities that were provided to expose the sample and the reference. The latter was an empty pan. The LED source was synchronized with the DSC by a shutter system placed between the fiber and the lens. It was controlled by an Arduino uno board programmed with the Arduino 1.05 program available from Arduino. The DSC software controls the event output of this device, which acts as a digital switch. This information went to the Arduino uno board, which controls the shutter in ON/OFF position by a servo motor. Reference[S6] provides more details.

Table S2. Kinetic traces of photo-DSC experiments taken with different sensitizers and coinitiators (**C1**, **C3**) under nitrogen. Experimental conditions according to Table 3 in manuscript.

| sensitizer | **C1** | **C3** |
| --- | --- | --- |
| **1** |  |  |
| **2a** |  |  |
| **2b** |  |  |
| **2c** |  |  |
| **3** |  |  |

Table S3. Kinetic traces of photo-DSC experiments taken with different sensitizers and coinitiators (**C1**, **C3**) under nitrogen. Experimental conditions according to Table 3 in manuscript.

| sensitizer | **C1** | **C3** |
| --- | --- | --- |
| **1** |  |  |
| **2a** |  |  |
| **2b** |  |  |
| **2c** |  |  |
| **3** | No response in the DSC upon exposure | No response in the DSC upon exposure |

## 6.2. DMA

The crosslinked polymer films were prepared under light exposure emitting at 365 nm, where a spacer of 80 mm was inserted to control the sample thickness. The solutions were applied on the glass substrate with a spacer and covered by another glass plate for exposure. The polymer films were then obtained after 15 minutes of irradiation with an intensity of 200 mW/cm^2^. The mechanical property analysis of the obtained polymer films was conducted using DMA Q800 from TA Instruments equipped with a linear tension clamp. The measurements were carried out with a constant frequency of 1 Hz, an amplitude of 10 mm as well as a preload of 0.01 N varying from -50°C to 150°C with a ramping rate of 3 °C/min where a sinusoidal force (stress) was applied to the samples and resulting in respective strain. With this oscillating loading, the mechanical properties measured could be reflected by the value of the storage modulus E´ and loss modulus E´´. Due to the large segments of the polymer, there is a phase lag or mechanical damping during the movement of polymer chains, which is known as tan𝛅 (tan𝛅 = *E*´´/*E*´). This can also be seen as a critical factor in describing the mechanical property. Therefore, as a comparison, the values of the glass transition *T*_g_ determined by the onset of storage modulus *E*´, the peak maximum of loss modulus E´´, and the peak maximum of tan𝛅 were displayed. Reference[S6] provides more details.


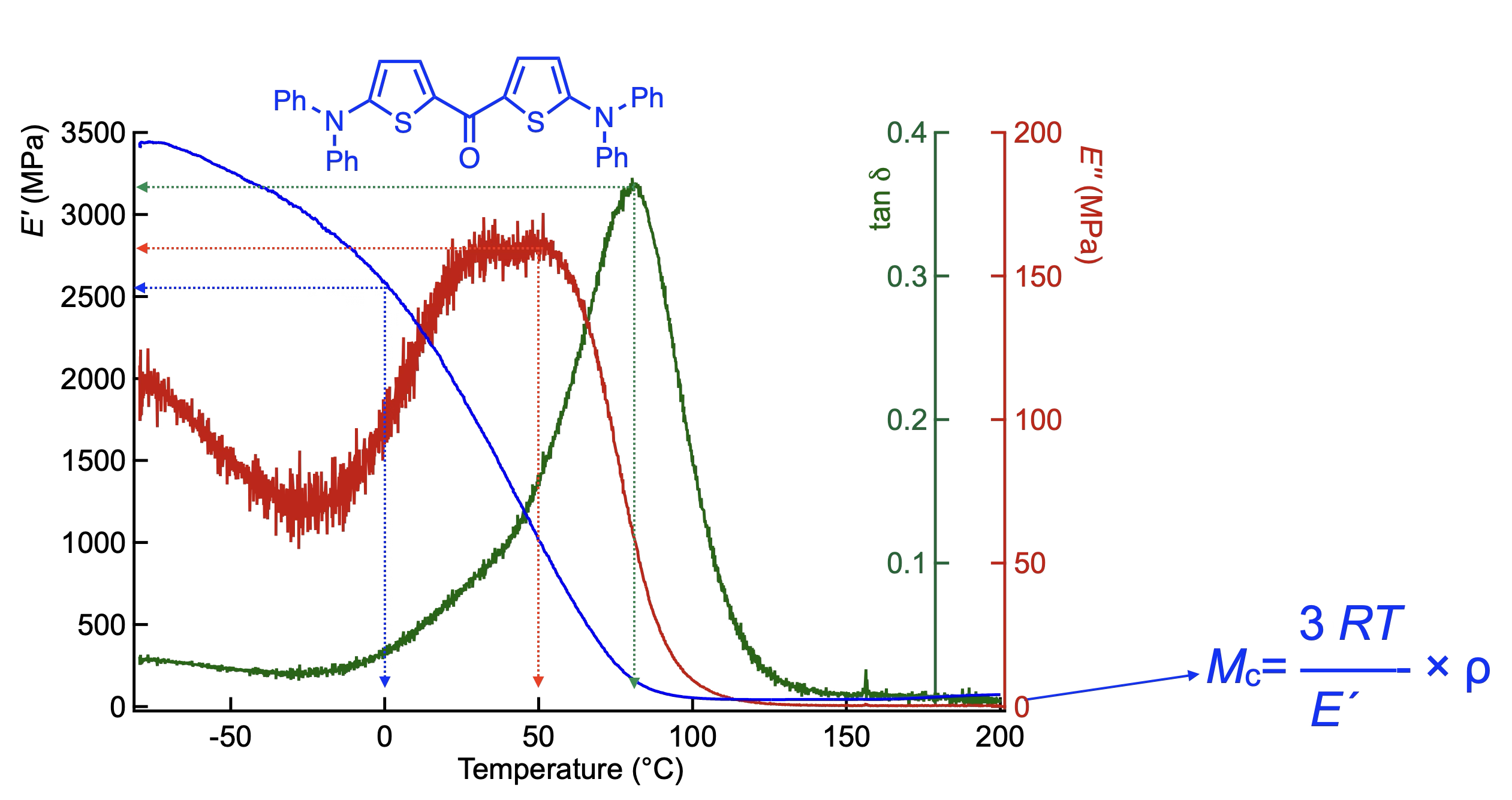


Figure S14: DMA of **2a** with the coinitiator **C1** in the monomer mixture TPGDA:UDMA= 2:3 conditions applying UV-LED as exposure source emitting at 395 nm. Experimental setup according to reference[S6].

# 7. Cytotoxic studies

Experiments were pursued by IWW Institute for Water Research gGmbH, Mülheim an der Ruhr, Germany. A machine translation (Deepl Pro) of the German report is part of this supporting information *vide infra*. The original German document can be obtained upon request by the authors.

To classify a substance as harmless, it is usually necessary to investigate its potential cytotoxic properties. It is therefore essential to select a standardized test procedure for these investigations. The following chapter describes the MTT test in more detail, which is evaluated in accordance with DIN EN ISO 10993-5 to obtain a validated test result.

For the MTT test (3-(4,5-dimethylthiazol-2-yl)-2,5-diphenyltetrazolium bromide), the sample substance is prepared as a saturated solution in DMSO. To rule out cytotoxic effects of the solvent, the sample solution is diluted by a factor of 1000. This results in a maximum test concentration of 0.136 mg/L. To achieve higher test concentrations, an additional dilution factor of 500 was used, resulting in maximum test concentrations of 0.272 mg/L. The formazan formed cannot leave the cell and is only released through cell lysis. It is then quantified photometrically at a wavelength of 595 nm. The amount of formazan detected is proportional to the number of metabolically active eukaryotic cells. Since living cells are required for the reduction of MTT, metabolic activity serves as a measure of cell damage, from which cytotoxicity can be derived. A reference sample without test substance (negative control) is used for the measurement. If the number of living cells falls below 70%, the test substance has cytotoxic potential. Cell lines from the ovaries of Chinese hamsters (CHO-9 cells) are used for the test procedure. These are seeded on 96-well microtiter plates and exposed to the test substance for 24 hours. In the next step, the cells are purified and mixed with MTT. After a two-hour incubation, cell lysis occurs, followed by photometric analysis to quantify the formazan.

**Cytotoxic report pursued by IWW Institute for Water Research**

# 7. ROHS Statement

# 8. References

[S1] H. Hartmann, J. Schumann, A. Kanitz, W. Rogler, "Preparation of diarylaminothiophenes as electroluminescent phosphors*"*, WO2001053287, (Siemens AG), **2001**.

[S2] A. Noack, A. Schöder, H. Hartmann, "Preparation and spectroscopic characterization of N,N'-persubstituted bis(2-amino-5-thienyl)thioketones and some of their aza analogues", *Phosphorus, Sulfur Silicon Relat. Elem.* **2001**, *176*, 185-190. 10.1080/10426500108055116.

[S3] A. Noack, H. Hartmann, "Synthesis and characterisation of N,N-disubstituted 2-amino-5-acylthiophenes and 2-amino-5-acylthiazoles", *Tetrahedron* **2002**, *58*, 2137-2146. 10.1016/s0040-4020(02)00083-2.

[S4] A. Noack, H. Hartmann, "Preparation and spectroscopic characterisation of some 5-methylmercapto- and 5-dimethylamino-substituted tris-(2-thienyl)methinium perchlorates", *Chem. Lett.* **2002**, 644-645. 10.1246/cl.2002.644.

[S5] T. Brömme, D. Oprych, J. Horst, P. S. Pinto, B. Strehmel, "New iodonium salts in NIR sensitized radical photopolymerization of multifunctional monomers", *RSC Advances* **2015**, *5*, 69915-69924. 10.1039/C5RA12236H.

[S6] X. Luo, X. Liu, H. Guo, R. Li, M. Wang, X. Li, S. Li, S. Liu, J. Li, V. Strehmel, Q. Wang, G. Yilmaz, K. Matyjaszewski, B. Strehmel, Z. Chen, "Biomass-derived carbon dots for the initiation of conventional radical and ATRP-based photopolymerization processes", *Nat. Protoc.* **2025**. 10.1038/s41596-025-01210-3.
